# The rebar repair for radial meniscus tears: a biomechanical comparison of a reinforced suture repair versus parallel and cross-stitch techniques

**DOI:** 10.1186/s40634-019-0206-4

**Published:** 2019-08-22

**Authors:** Patrick Massey, Kaylan McClary, David Parker, R. Shane Barton, Giovanni Solitro

**Affiliations:** Department of Orthopaedic Surgery, Louisiana State University Health Sciences Center- Shreveport, 1501 Kings Highway, Shreveport, LA 71103 USA

**Keywords:** Meniscus repair, Radial tear, Reinforcement technique

## Abstract

**Background:**

Radial meniscus tears can cause the meniscus to be completely incompetent. This serious type of meniscus tear can be difficult to repair. Techniques have been developed that juxtapose the meniscus tear edges and are able to withstand high loads. The purpose of this study was to determine the load to failure of a reinforced suture bar repair (Rebar Repair) for radial meniscus tear and compare it to the parallel suture technique and cross-stitch technique and to compare mode of failure among all three groups. The hypothesis was that the Rebar Repair will have a higher load to failure than both the parallel technique and the cross-stitch technique and that the Rebar Repair would have a lower rate of suture cutting through the meniscus.

**Methods:**

Forty-eight menisci were tested from 24 human knee specimens, with 16 menisci in each group evenly distributed between medial and lateral menisci. Radial mid body meniscal tears were recreated and repaired with one of three inside-out techniques: the 2-parallel suture technique, 2 cross-stitch sutures, and the Rebar Repair. The specimens were cycled between 5 N to 30 N and axially loaded to failure perpendicularly across the repair site.

**Results:**

The average load to failure of the parallel group, cross-stitch group and Rebar Repair was 85.5 N ± 22.0, 76.2 N ± 28.8 and 124.1 N ± 27.1 respectively. The Rebar Repair had a higher load to failure than the parallel group (*p* < 0.01) and cross-stitch group (*p* < 0.01). There was no difference in the load to failure between the cross-stitch and parallel group (*p* = 0.49). The failure mechanism was different when comparing the 3 groups (*p* < 0.01). The predominant mode of failure for both the parallel and cross-stitch group was suture cutout through the meniscus (88% and 94% respectively). The Rebar Repair failed due to suture rupture in 50% and suture cutout through the meniscus in 50%.

**Conclusion:**

The Rebar Repair for radial meniscus tear has a higher load to failure and a lower rate of suture cutout through the meniscus than the parallel technique and cross-stitch technique.

**Clinical relevance:**

Radial meniscus tears lead to decreased hoop stresses of the meniscus and effectively a non-functional meniscus. Newer techniques may have a higher load to failure leading to more successful repairs.

## Introduction

Prior to the 1990s, radial meniscus tears were ignored or treated with meniscectomy (Newman et al. 1993). Previous studies have shown that radial meniscus tears are much more detrimental than other types of meniscus tears such as longitudinal or horizontal tears with respect to decreased contact area and increased contact pressure (Mononen et al. 2013; Ode et al. 2012). Radial tears in particular are difficult to repair and with concern to have poor healing rates due to injury of avascular or white/white zones (Barber-Westin and Noyes 2014). They can be compared to complete meniscus root avulsions as radial meniscus tears lead to decreased hoop stresses of the meniscus and effectively a non-functional meniscus. This incompetent meniscus can lead to early arthritis, which has led to an effort to repair these challenging tears (Bin et al. 2004; Lee et al. 2011). Although a challenging type of tear to repair, a radial tear is an important type of tear to repair.

The trend toward radial tears has been shifting towards repair (Abrams et al. 2013). Newer techniques have demonstrated some success with repair of the radial meniscus tear with promising results seen both biomechanically and clinically (Beamer et al. 2015; Bhatia et al. 2016; Branch et al. 2015; Buckley et al. 2019; Choi et al. 2010; Haklar et al. 2008; Lee et al. 2012; Matsubara et al. 2012; Milchteim et al. 2016; Nakata et al. 2011; Noyes and Barber-Westin 2012; Stender et al. 2018; Bedi et al. 2010). The standard technique used has classically been the parallel stitch, but recently a cross stich method has been suggested to outperform the cross stitch (Matsubara et al. 2012). These still do not reach normal meniscus strength (Bhatia et al. 2016; Branch et al. 2015; Matsubara et al. 2012; Milchteim et al. 2016). Despite this trend towards improvement in outcomes, we continue to seek repair methods that more securely and reliable restore meniscal functional anatomy. Recently, a reinforced suture bar (Rebar) repair was developed with the goal of reinforcing the repair to protect the repair and allow for healing. The purpose of this study was to determine the load to failure of a reinforced suture bar repair (Rebar Repair) for radial meniscus tear and compare it to the parallel suture technique and cross-stitch technique and to compare mode of failure among all three groups. The hypothesis was that the Rebar Repair would have a higher load to failure than both the parallel technique and the cross-stitch technique and that the Rebar Repair would have a lower rate of suture cutting through the meniscus.

## Methods

### Specimen preparation

This study did not require Institutional Review Board approval, as under current guidelines, it is a cadaver biomechanical study. Forty-eight menisci were tested from 24 fresh frozen human cadaver knee specimens. The knees were thawed for 24 h and all soft tissue was dissected from the tibia except for the medial and lateral meniscus and meniscal attachments to the tibia. The menisci were inspected by a board certified sports medicine fellowship trained orthopaedic surgeon and excluded if there was a meniscus tear. Each tibia was cut transversely 6 in. distal to the joint line. A 3-in.-long, 3/16 diameter stainless steel rod was placed medial to lateral in the tibia and it was potted in polyester resin (3 M Bondo, Maplewood, MN) inside of metal boxes (3.5x3x2in). The medial and lateral meniscus of each knee was measured for meniscus width, length, mid-body width and thickness, and the data was recorded. Eighteen of the specimens were matched cadaver pairs, while the other 6 were matched left and right knees from different donors. The 3 different cadaver pairs were paired based on similar meniscus size. The knees were randomly chosen for each series. Three series of paired testing were performed with 8 knees (16 menisci) tested per series. For the parallel group, cross-stitch group, and Rebar group, there were 8 knees in each group: 4 left and 4 right, medial and lateral meniscus repaired with the same technique. This allocated 16 menisci in each of the three groups (48 menisci total).

After dissection, a full radial meniscus tear, including tear of the coronary ligament, was created at the mid-body, using a #10 scalpel blade. The repair was performed in one of three techniques: the parallel stitch, the cross stitch and the Rebar Repair. These techniques were performed with Arthrex 2–0 FiberWire Meniscus Needles (Arthrex, Naples, Florida) in an inside out technique. This was done in an open fashion with arthroscopic instruments. All sutures were tied over the outer surface of the meniscus by the senior author with seven alternating half hitches.

Upon completion of the repair, the anteromedial root of the medial meniscus was resected from the medial plateau and the posterolateral root of the lateral meniscus was resected from the lateral plateau. The coronary ligament was cut from the free end of the meniscus up to the site of the tear to ensure all force applied would be translated across the repair site.

### Repair techniques

**Fig. 1:**
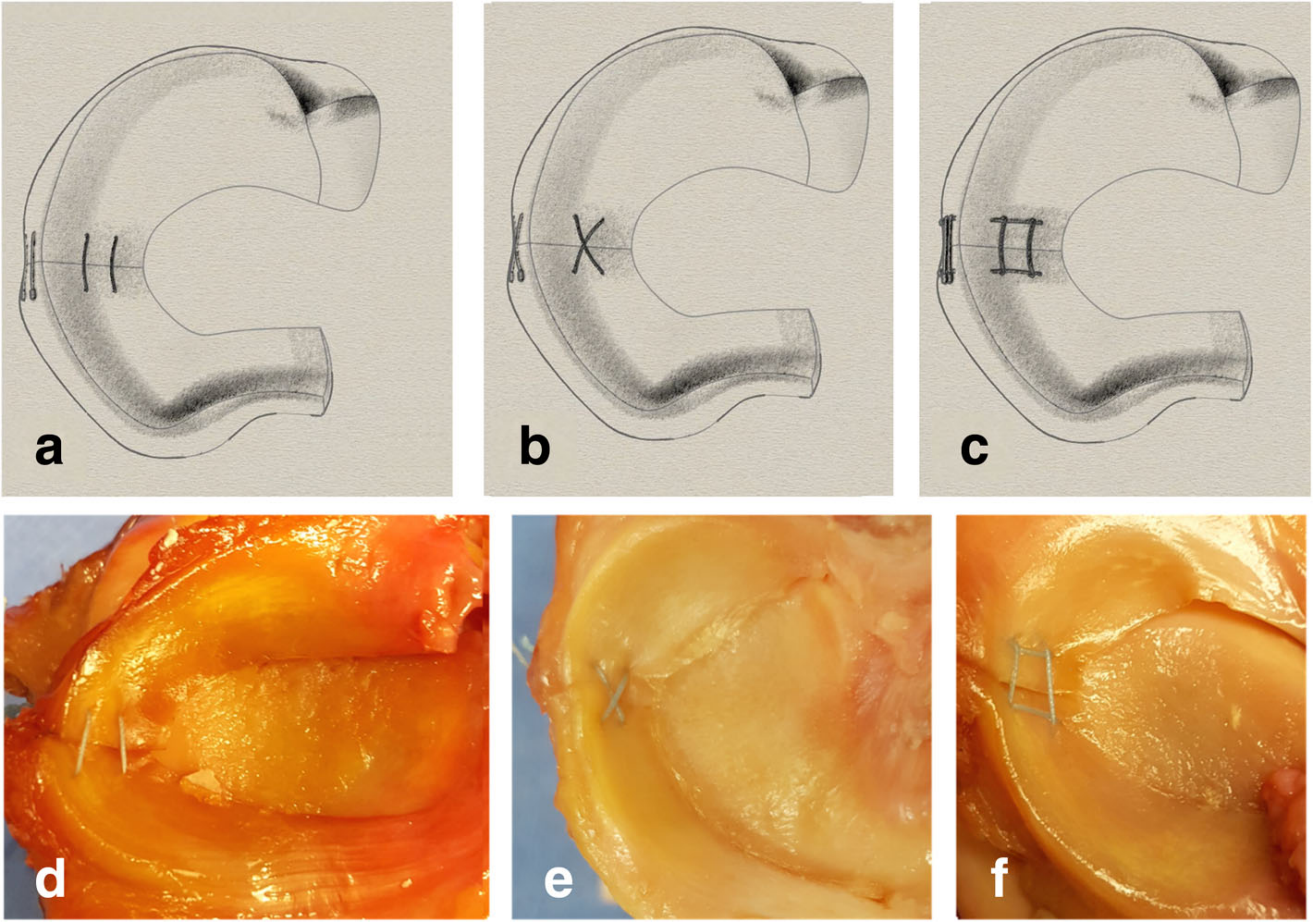
**a** Standard Repair with 2 parallel sutures across the radial tear. **b**. Cross Stitch Repair with 2 perpendicular sutures in a “X” configuration. **c**. Rebar Repair with 4 sutures, 2 longitudinal reinforcing sutures on each side of the tear with 2 horizontal stitches across the reinforcing sutures and the tear. **d**, **e**, **f** are cadaver views of the parallel suture repair, cross stitch repair and Rebar Repair respectively

Parallel Stitch (See Fig. 1a): Two 2.0 FiberWire meniscus needle sets were used. The first needle set was passed 5 mm from the inside of the meniscus and 5 mm from radial tear on either side of the tear through the outer surface of the meniscus. The next needle set was passed 5 mm from the first stitch towards the capsular side, maintaining 5 mm from the radial tear. The needles were then cut off and all suture pairs tied.

Cross Stitch (See Fig. 1b): Two 2.0 FiberWire meniscus needles sets were used with the same template and the technique described by Matsubara (Matsubara et al. 2012). For the first set, one meniscus needle was inserted 5 mm from the inner meniscus and 5 mm from the radial tear then exited the meniscus laterally then the other needle was placed obliquely 10 mm from the inner meniscus edge and 5 mm from the radial repair on the opposite side from the first pass. The next set of needles was inserted in a similar fashion in the opposite direction to create an “X configuration”. The needles were then cut off and all suture pairs tied.

**Fig. 2:**
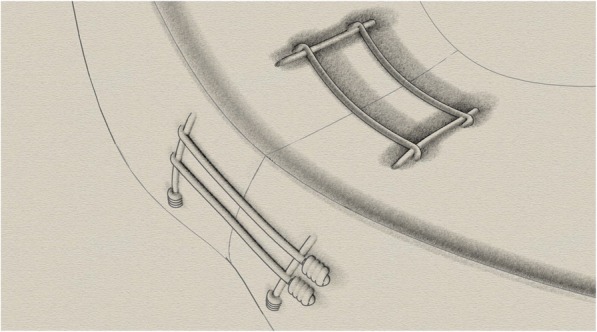
Rebar Repair illustration. The 2 horizontal sutures are placed exactly juxtaposed to the vertical sutures. The horizontal sutures are running perpendicular to the radial meniscus tear

Rebar Repair (See Fig. 1c): Four needle sets were used. First, a vertical mattress type repair or reinforcing stitch was performed by placing the first needle 2 mm from the inner meniscus (central) and 5 mm posterior to the tear, then the second needle 8 mm from the inner meniscus and 5 mm posterior to the tear. On the other side of the tear, 5 mm anterior from the tear, another vertical mattress suture was passed with the next needle set in a similar fashion. The parallel horizontal sutures were then passed while making sure that each needle passed on the side of the reinforcing stitch away from the tear (see Fig. 2). Two 2.0 FiberWire meniscus needle sets were used. The first needle set was passed 7 mm from the inner meniscus and 5 mm from radial tear on each side of the tear through the more peripheral surface of the meniscus. The next needle set was passed 3 mm from the inner meniscus (central) and 5 mm anterior and posterior from the radial tear on each side of the tear. Each parallel suture was directly juxtaposition to a vertical suture. The needles were then cut off and all suture pairs tied.

### Testing

**Fig. 3:**
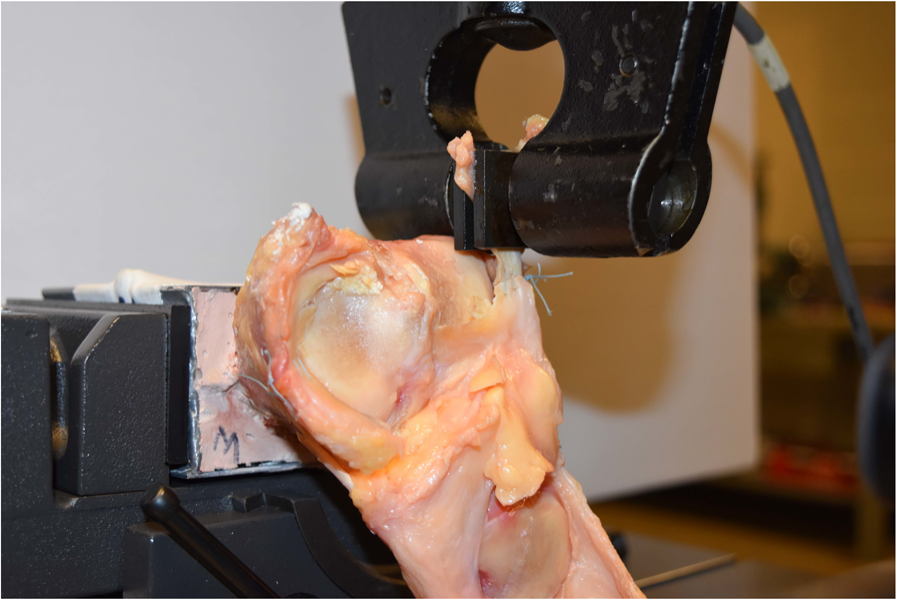
Lateral view of Instron testing for left cadaver knee lateral meniscus. A hydraulic clamp is applied to the posterior horn with the tibia potted and anterior horn and capsular attachments maintained

Mechanical testing was performed attaching the femur to the actuator of a servohydraulic testing system (Model 8874, Instron, Canton, MA). For each test, the meniscus root (anteromedial for medial meniscus and posterolateral for lateral meniscus) was placed into the Instron pneumatic clamps with the potted tibia stabilized with additional clamps. (See Fig. 3).

Tensile force was applied along the free meniscal end. The sample was then cyclically loaded between 5 N and 30 N at 1 Hz for 500 cycles. The meniscus was pre-loaded for 50 cycles; then the displacement was recorded as the distance the Instron clamp translated after 500 cycles. Next the sample was loaded until failure at a rate of 5 mm/minute. Failure was determined to be the force at which the force value dropped to 80% of the maximum load. The energy to failure was calculated as the area under the force -displacement curve until the failure load. After testing, the specimens were inspected for failure modes, which included suture pull out from the meniscus vs. rupture of the suture knot.

### Statistics

Based off of a recent study using the same suture material and comparing 2 parallel sutures to the cross stitch an a-priori power analysis was performed (Milchteim et al. 2016). It was determined that the number needed for each group was 9 in order to obtain a power of 0.8. The values used for calculation were 89.6 for μ_1_, 16.3 for SD_1_, 72.1 for μ_2_ and 11.7 for SD_2 (__Rosner_ 2000_)_.

The three groups were compared with multiple statistical methods. The dimensions of the three groups menisci were compared using an ANOVA. The categorical data such for sex distribution of the cadavers in each group and mode of failure were compared using a chi-square. The biomechanical data was compared using an ANOVA; and group comparisons were made with Tukey’s honestly significant difference (hsd) post-hoc testing. The overall data was analyzed as well as biomechanical data specific to each meniscus.

## Results

There were 48 menisci tested with 16 specimens in each of the three groups. A post-hoc power analysis revealed a 1 – β of .997. The overall average age of the cadavers was 65.1 yr. ± 8.5. The distribution of the cadaver demographics is outlined in Table 1.

**Table 1:** Demographic data of cadaver specimens

Group	Age (years)	Weight (kg)	Height (cm)	BMI (kg/m3)	Sex	
					M	F
Parallel	66.8	60.2	170	21.8	2	6
Cross	67.1	54.7	166	20.0	3	5
Rebar	61.4	60.7	170	21.6	3	5
P Value	0.15	0.60		0.83	0.83	

There was no difference in the age of each of group, with average age for the parallel, cross-stitch and Rebar Repair group of 66.8 yr. ±11.2, 67.1 ± 7.9, and 61.4 yr. ±5.1, respectively (*p* = 0.15). The average weight of the parallel, cross-stitch, and Rebar Repair cadavers was 60.2, 54.7, and 60.7 kg respectively, with no difference between groups (*p* = 0.60). There was also no difference in the height of the cadavers between the three groups (*p* = 0.462) with an average height of 170, 166, and 170 cm for the parallel, cross-stitch, and Rebar Repair cadavers respectively. The average Body Mass Index of the parallel, cross-stitch, and Rebar Repair cadavers was 21.8, 20.0, and 21.3 kg/m^2^ respectively, with no difference between groups (*p* = 0 .83). The was no difference in the distribution of sex by group with the parallel, cross-stitch and Rebar Repair group having 2 males, 6 females, 3 males, 5 females and 3 males, 5 females respectively (*p* = 0.83). The lateral meniscus and medial meniscus dimensions were not different between the three groups (See Tables 2 and 3).

**Table 2:** Lateral Meniscus dimensions (mm) of 3 groups: parallel stitches, Cross Stitch and Rebar Repair

Group	Width	sd	Length	sd	Mid-Body Width	sd	Thickness	sd
Parallel	29.3	(4.3)	32.9	(3.8)	9.3	(1.5)	5.6	(1.2)
Cross	28.9	(4.1)	32.1	(5.7)	8.8	(3.2)	5.3	(1.5)
Rebar	30.6	(3.6)	32.0	(4.0)	10.6	(3.4)	5.1	(1.2)
P-value	0.90		0.50		0.11		0.82

**Table 3:** Medial Meniscus dimensions (mm) of 3 groups: parallel stitches, Cross Stitch and Rebar Repair

Group	Width	sd	Length	sd	Mid-Body Width	sd	Thickness	sd
Parallel	27.9	(4.1)	41.3	(4.8)	9.0	(1.7)	4.7	(1.5)
Cross	27.4	(3.5)	38.5	(5.0)	7.6	(1.4)	4.9	(1.0)
Rebar	26.9	(3.1)	40.9	(5.4)	8.4	(1.6)	4.7	(1.3)
P-value	0.83		0.89		0.89		0.53	

**Fig. 4:**
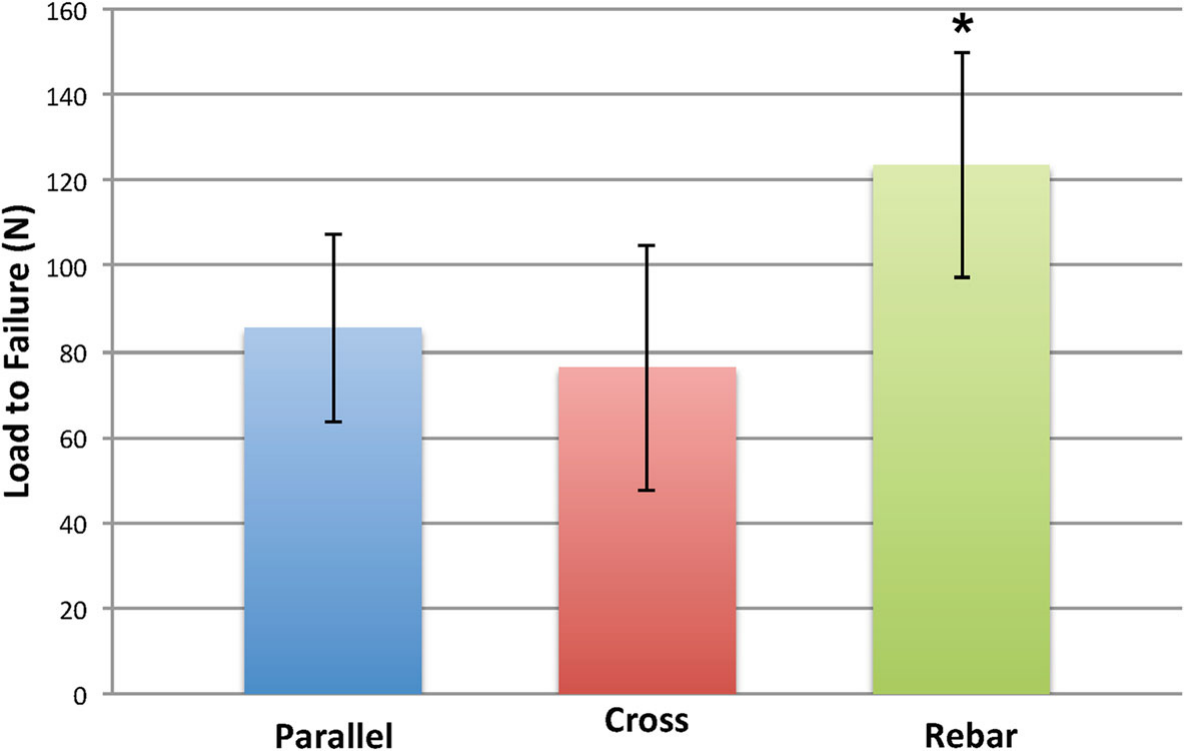
Bar graph showing average load to failure values (N) for all three repair types with standard deviation error lines. * Load to failure was significantly higher for the Rebar Repair than the parallel and cross-stitch groups (*p* < 0.01)

The average load to failure of the parallel, cross-stitch and Rebar Repair was 85.5 N ± 22.0, 76.2 N ± 28.8 and 124.1 N ± 27.1 respectively (see Fig. 4).

Overall, there was a difference in the load to failure among the Rebar Repair, parallel group and cross-stitch group (*p* < 0.01). The Rebar Repair had a higher load to failure than the parallel group (*p* < 0.01) and cross-stitch group (*p* < 0.01). There was no significant difference in the load to failure between the cross-stitch and parallel group (*p* = 0.49). There was no significant difference between all 3 techniques with respect to stiffness (*p* = 0.66). There was a higher energy until failure for the Rebar Repair versus the parallel and cross-stitch groups (*p* < 0.01, *p* < 0.01 respectively). There was no difference in the energy until failure between the parallel group and cross-stitch group (*p* = 0.08). When evaluating the medial and lateral meniscus separately, there was a difference in load to failure between the three groups (see Tables 4 and 5).

**Table 4:** Lateral Meniscus Biomechanical Data of 2 parallel stitches, Cross Stitch and Rebar Repair

Group	Load to Failure (N)	SD(N)	Stiffness (N/mm)	SD(N/mm)	Energy to Max Load (J)	SD (J)
Parallel	82.2	13.6	19.0	3.55	0.87	0.25
Cross	62.9	20.4	17.8	4.76	0.55	0.28
Rebar	122.0	26.8	20.1	2.60	1.78	0.85
P-value	<0.01		0.50		<0.01

**Table 5:** Medial Meniscus Biomechanical Data of 2 parallel stitches, Cross Stitch and Rebar Repair

Group	Load to Failure (N)	SD(N)	Stiffness (N/mm)	SD(N/mm)	Energy to Max Load (J)	SD (J)
Parallel	88.7	28.8	20.7	1.90	0.94	0.43
Cross	89.5	30.9	19.8	5.60	0.80	0.44
Rebar	126.5	27.5	18.6	2.19	2.04	1.47
P-value	0.03		0.49		0.02	

There was no difference in displacement between the 3 groups after 500 cycles (*p* = 0.9). The parallel, cross-stitch, and Rebar Repair had mean displacements of 1.83 mm ± 2.18, 1.70 mm ± 2.05, and 1.50 mm ± 1.76 respectively.

**Fig. 5:**
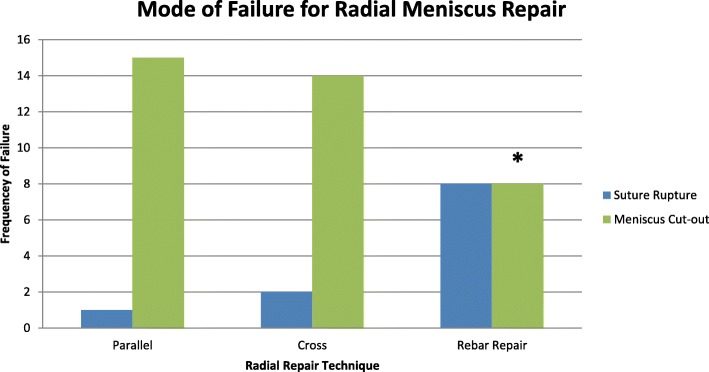
Bar graph of the different modes of failure. * There was less meniscus suture cutout in the Rebar Repair group compared to the parallel and cross-stitch groups (*p* < 0.01). The predominate mode of failure in the cross-stitch group and parallel repair group was meniscus suture cutout. There were 8 failures due to suture rupture and 8 due to meniscus suture cutout in the Rebar Repair group

The mode of failure was different between the 3 groups (*p* < 0.01). The predominate mode of failure in the parallel and cross-stich group was meniscus cutout (94% and 75% respectively). The Rebar Repair failed equally due to suture rupture and suture cutout (50% each). The frequency for all modes of failure between each group can be seen in Fig. 5.

## Discussion

The current study demonstrated the Re-bar Repair has a higher load to failure than the parallel repair and cross-stich repair for radial meniscus tear. There was no difference in the stiffness or displacement between all 3 groups. Additionally, it was found that there was a lower rate of suture cutout through the meniscus.

Clinically this reinforced repair may be a useful tool in repair of these difficult radial tear types. A similar type of reinforced repair has clinically shown an 89% success rate at 18 months post-operatively and high rate of healing on second look arthroscopy (Nakata et al. 2011; Tsujii et al. 2018). This radial repair called the “Tie-Grip” suture utilized 3 or 4 horizontal sutures and a different suture material than the Rebar Repair, but has shown that re-inforced repairs can have promising clinical results (Nakata et al. 2011).

In an effort to determine the best technique for repairing radial meniscus tears, several previous biomechanical studies have analyzed different repair techniques. Matsubara compared a 2-horizontal parallel stitch versus cross configuration in 2 groups of 20 fresh human menisci for radial meniscus tear (Matsubara et al. 2012). That study showed a significant increase in load to failure (78.96 ± 19.27 N vs. 68.16 ± 12.92 N; *p* < 0.05) for the cross-stitch repair versus the parallel group. The authors also showed higher stiffness with the cross-stitch repair versus parallel repair (8.01 ± 1.54 N/mm vs. 6.46 ± 1.12 N/mm; *p* < 0.05). Conversely, our study showed no significant difference between the cross and parallel stitch. The load to failure values for the parallel and cross-stitch reported by Matsubara were, however similar to the current study. In previous studies, pneumatic grips were used on both roots which may have led to slippage (Branch et al. 2015; Matsubara et al. 2012; Milchteim et al. 2016).

The native intact meniscus can have variable mechanical properties. Yan et al. performed biomechanical studies on the intact human meniscus divided into 4 semilunar segments and found the range of the ultimate tensile load for each segment to be 22.54 N, 67.59 N, 179.56 N and 212.03 N from the inner to outer segments (Yan et al. 2016). The current study showed the Rebar Repair to have comparable failure loads to the native intact meniscus.

To reduce the effect of slippage, our menisci were tested in the manner demonstrated by Bhatia et al. with tibia potting and retention of meniscal attachments (Bhatia et al. 2016). In their study, the authors compared a double parallel suture vs. double parallel suture anchored with transtibial 2-tunnel cross repair. The results showed increase in failure load of 196 N versus 106 N (*p* = 0.004) with the transtibial stitch versus the parallel stich. The resultant values in the study were even higher than the results in the current study which may be explained by a difference in testing methods. For the current study, the coronary ligament attachment to the section of meniscus being pulled was released to remove the contribution of the ligament to the tensile strength. However, in the study by Bhatia, it appears the capsular attachment was left intact (Bhatia et al. 2016). Although the transtibial stitch did show significant increase in ultimate failure load, the authors did comment that the technique was more technically challenging as well as required additional instrumentation.

Additional studies have compared all-inside versus inside-out techniques for radial meniscus. Lee et al. compared an all-inside Z configuration versus 2 parallel horizontal inside out sutures, using porcine menisci (Lee et al. 2012). They showed no significant difference between load to failure with all-inside versus inside out (86.2 N vs. 89.8 N respectively, *p* = 0.69). While the authors used porcine menisci, their load to failure for the 2 parallel sutures was similar to our study.

Additionally, Branch et al. performed a study which compared the 2 parallel stitch inside out technique with an all inside parallel technique, an all inside cross stitch with single horizontal stitch and an all inside described “Mason Allen” technique (Branch et al. 2015). This study showed no difference between load to failure for the all inside “mason allen” technique versus all-inside horizontal (86 N vs. 75 N respectively). These results are comparable to the load to failure for the parallel repair in the current study, but appear to be lower than the load to failure of the Rebar Repair (Branch et al. 2015). While the all-inside “mason allen” technique may be similar to the current technique, it is performed all-inside which may be technically challenging (Branch et al. 2015). Also, the Rebar Repair is an inside-out technique, placing the knots on the outside of the meniscus or capsule, which may improve biomechanical performance. Some studies have also evaluated trans-tibial suture repairs for radial meniscus repair. These studies have utilized sutures pulled through the meniscus and anchored over a button on the tibia (Bhatia et al. 2016). Other trans-tibial studies have shown the addition of horizontal sutures prevented meniscus cut-out similar to the findings the Rebar Repair (Buckley et al. 2019).

A more recent study has compared the hashtag, cross-tag and cross suture techniques showing load to failure of 86.08 N, 62.50 N, and 81.43 N, respectively. The authors found no significant difference with load to failure between all three techniques. The authors also found stiffness for the hashtag, cross-tag and cross suture techniques to be 9.30, 8.13 N/mm, and 8.48 N/mm. The current study shows similar failure loads for the cross suture repair. The Rebar Repair in our study, however showed higher load to failure than the cross suture and parallel suture techniques. This may be due to the fact that the Rebar Repair uses horizontal sutures which are placed directly adjacent to the vertical sutures. The hashtag repair previously reported is shown in figures to have horizontal sutures placed further from the vertical suture limbs (Stender et al. 2018). Additionally, the previous study used different suture material than the current study making a direct comparison difficult. The predominant mode of failure was also different for the hashtag previously reported, with 83% of hashtag repairs failing from tissue failure. While the hashtag mainly failed due to tissue failure, the current study showed a mix of suture breakage and tissue failure for the Rebar Repair (Stender et al. 2018). The Rebar Repair has a lower rate of tissue failure or meniscus cutout, which may be due to the repair spreading stress across a larger surface area. Instead of the repair ripping out of the meniscus, the Rebar Repair may spread the stress over a larger surface area until the suture fails at higher loads.

## Limitations

One limitation for the study was the average age of specimens utilized from cadavers aged around 65 years of age. This age is often considered beyond the age of meniscal repair. While the age of specimens was older, only intact menisci were used for this study, and there was no difference in anatomical morphology between all three groups with respect to the meniscus dimensions. Another limitation is that the repair techniques were done open. While the techniques used were performed using arthroscopic tools, an arthroscopic surgery could affect the precision of needle placement. In order to increase consistency this open technique with arthroscopic instruments was performed, but in the future, arthroscopic studies should be done. Also, it should be noted that this is a biomechanical study only comparing one element of testing of the menisci in form of tensile strength. The meniscus also experiences shear and compressive forces not accounted for in this study. Furthermore, our study does not address gapping at the site of the meniscal tear. The displacement recorded is the distance the Instron clamp moved after preloading with 50 cycles. It also does not address the ability of the meniscus to heal. There is the possibility that adding more stitches compresses the menisci affecting the already limited blood supply. Future animal studies are warranted to further evaluate performance in vivo.

## Conclusion

The Rebar Repair for radial meniscus tear has a higher load to failure and a lower rate of suture cutout through the meniscus than the parallel technique and cross-stitch technique.

## Data Availability

The datasets used and/or analysed during the current study are available from the corresponding author on reasonable request.
